# Online Japanese-Language Information on Lifestyle Factors Associated With Reduced Fertility: Content Analysis

**DOI:** 10.2196/19777

**Published:** 2020-08-25

**Authors:** Rie Yokota, Tsuyoshi Okuhara, Haruka Ueno, Hiroko Okada, Emi Furukawa, Takahiro Kiuchi

**Affiliations:** 1 Department of Health Communication Graduate School of Medicine The University of Tokyo Bunkyo-ku, Tokyo Japan; 2 Department of Health Communication School of Public Health The University of Tokyo Bunkyo-ku, Tokyo Japan; 3 Department of Health and Dietetics Faculty of Health and Medical Science Teikyo Heisei Unievrsity Toshima-ku, Tokyo Japan

**Keywords:** content analysis, online information, lifestyle factor, fertility, infertility, reproductive health

## Abstract

**Background:**

Approximately one-third of Japanese couples currently worry or previously worried about infertility. To develop strategies for the primary prevention of infertility as a population approach, it is important for the general population to be knowledgeable about fertility and infertility. The internet may contribute to the dissemination of information regarding infertility and fertility. However, few studies have examined online information about fertility.

**Objective:**

This study aimed to quantitatively examine online Japanese-language information about lifestyle factors associated with reduced fertility.

**Methods:**

We conducted online searches, using the 10 search terms with the highest numbers of searches that people hoping to conceive are likely to input in two major search engines in Japan (Google Japan and Yahoo! Japan). From the 2200 retrieved websites, 1181 duplicates and 500 websites unrelated to our objective were excluded, resulting in a final dataset of 519 websites. Coding guidelines were developed for the following lifestyle factors associated with reduced fertility: sexually transmitted diseases, psychological stress, cigarette smoking, alcohol use, nutrition and diet, physical activity and exercise, underweight, overweight and obesity, and environmental pollutants.

**Results:**

In terms of the website author’s professional expertise, 69.6 % of the coding instances for the selected lifestyle factors were mentioned by hospitals, clinics, or the media, whereas only 1.7% were mentioned by laypersons. Psychological stress (20.1%) and sexually transmitted diseases (18.8%) were the most frequently mentioned lifestyle factors associated with reduced fertility. In contrast, cigarette smoking, alcohol use, nutrition and diet, physical activity and exercise, underweight, overweight and obesity, and environmental pollutants were mentioned relatively infrequently. The association between reduced fertility and sexually transmitted diseases was mentioned significantly more frequently by hospitals and clinics than by the media (*P*<.001). The association between reduced fertility and nutrition and diet was mentioned significantly more frequently by the media than by hospitals and clinics (*P*=.008). With regard to the sex of the target audience for the information, female-specific references to psychological stress, sexually transmitted diseases, nutrition and diet, underweight, physical activity and exercise, and overweight and obesity were significantly more frequent than were male-specific references to these lifestyle factors (psychological stress: *P*=.002, sexually transmitted diseases: *P*<.001, nutrition and diet: *P*<.001, underweight: *P*<.001, physical activity and exercise: *P*<.001, overweight and obesity: *P*<.001).

**Conclusions:**

Of the lifestyle factors known to be related to reduced fertility, cigarette smoking, alcohol use, and male-specific lifestyle factors are mentioned relatively infrequently in online information sources in Japan, and these factors should be discussed more in information published on websites.

## Introduction

### Background

At the 1994 United Nations International Conference on Population and Development, reproductive health was defined as “a state of complete physical, mental and social well-being and not merely the absence of disease or infirmity, in all matters relating to the reproductive system and to its functions and processes” [1]. Infertility is increasingly acknowledged as a global public health issue by the World Health Organization [2], and reproductive health implies that “people are able to have the capability to reproduce and the freedom to decide if, when and how often to do so” [1]. To aid decision making concerning fertility and assist the reproductive-aged population in optimizing their fertility and reproductive health, knowledge of the lifestyle factors associated with reduced fertility is crucial [3,4].

Infertility is defined as the failure to achieve conception following at least 12 months of unprotected sexual intercourse [5]. Infertility affects as many as 186 million people worldwide [6], and about 10% to 15% of couples experience infertility [7]. In 2015, as many as one in three Japanese couples (approximately 35.0%) reported currently or previously worrying about infertility, and more than one-sixth (approximately 18.2%) reported currently or previously undergoing screening for infertility or trying to achieve pregnancy through assisted reproduction technologies [8].

Numerous factors may contribute to reduced fertility in men and women. In addition to genetic background and reproductive history, environmental factors and current lifestyle habits have been proposed as causes of male and female infertility [9]. According to large systematic reviews and meta-analyses, the lifestyle factors associated with reduced fertility are (1) sexually transmitted diseases, (2) psychological stress, (3) cigarette smoking, (4) alcohol use, (5) nutrition and diet, (6) physical activity and exercise, (7) underweight, (8) overweight and obesity, and (9) environmental pollutants [10-27]. Given the situation of one-third of Japanese couples currently or previously worrying about infertility, for those who are trying to conceive or hope to have a child in the future, knowledge about the lifestyle factors associated with reduced fertility may help to prevent infertility.

A recent study found that about 60% to 70% of the reproductive-aged population in Japan responded incorrectly to a question about the association between cigarette smoking and reduced fertility [28]. Similarly, when asked about the association between female overweight and reduced fertility, approximately 80% to 90% of the reproductive-aged population in Japan answered incorrectly [28]. These findings indicate a lack of knowledge about the associations between reduced fertility and lifestyle factors such as smoking cigarettes and overweight and obesity among the reproductive-aged population in Japan.

In terms of sex differences, several studies have shown that, compared with women, men are less knowledgeable regarding the associations between lifestyle factors and reduced fertility [3,29]. Although most men (88.5%) regarded themselves as knowledgeable on this topic, only half of the men (53.1%) participating in a population-based survey were able to identify the lifestyle factors associated with reduced fertility [4]. A recent survey in Japan found that only 46.4% of reproductive-aged men and 56.7% of reproductive-aged women were knowledgeable about male infertility factors, which constitute approximately 50% of infertility cases [30]. Moreover, according to the same survey, 38.0% of men reported that they did not intend to undergo a semen examination at a medical institution because they thought they had no infertility problems [30]. Because infertility is presumed to be a women’s issue [31], the reproductive-aged population has relatively low knowledge about issues related to male fertility [32].

Currently, the internet is a preferred and common source of health information [33]. About 72% of internet users access health-related information via the web [32]. Health information is conveyed to targeted audiences to influence their attitudes or behaviors [34], and the presentation of information and framing used in media portrayals affect the general public’s understanding of lifestyle factors associated with reduced fertility [35]. Thus, online information about fertility influences the general population’s knowledge about conception.

### Prior Work

Previous studies regarding information on infertility and fertility in the media have examined (1) newspaper reports about assisted reproductive technology [35]; (2) information on clinic websites [36]; (3) the readability, suitability, and quality of online information [32,37]; (4) online videos made by laypersons and informational infertility-related educational videos [38]; and (5) online emotional support and social media on infertility-seeking to reduce isolation [39,40]. As mentioned above, although people who are trying to conceive tend to have relatively low levels of knowledge about lifestyle factors associated with reduced fertility, especially male infertility, to our knowledge, few studies have investigated the content of online information on the lifestyle factors associated with reduced fertility.

People who are diagnosed with infertility and those receiving infertility treatment may receive accurate information on fertility from health care professionals. However, no studies have examined what kinds of online content are accessible to the general population, although online information is especially important for people who do not visit medical institutions but are trying to conceive or hoping to have a child in the future. Thus, it is crucial to examine online information on the lifestyle factors associated with reduced fertility.

### Goal of the Study

The study aimed to (1) quantitatively examine the information on lifestyle factors associated with reduced fertility accessible to people hoping to have a child who seek information regarding fertility on the internet and (2) identify the characteristics of this information in terms of lifestyle factors, the webpage author’s professional expertise, and the sex of the target audience (ie, information for men, women, or both).

## Methods

### Search Engine

We used a Japanese-language search string input into the two most popular search engines in Japan, Google Japan [41] and Yahoo! Japan [42]. Google Japan and Yahoo! Japan accounted for roughly 75% and 19% of all internet searches, respectively, at the end of October 2019 [43].

### Search Terms

**Figure 1:**
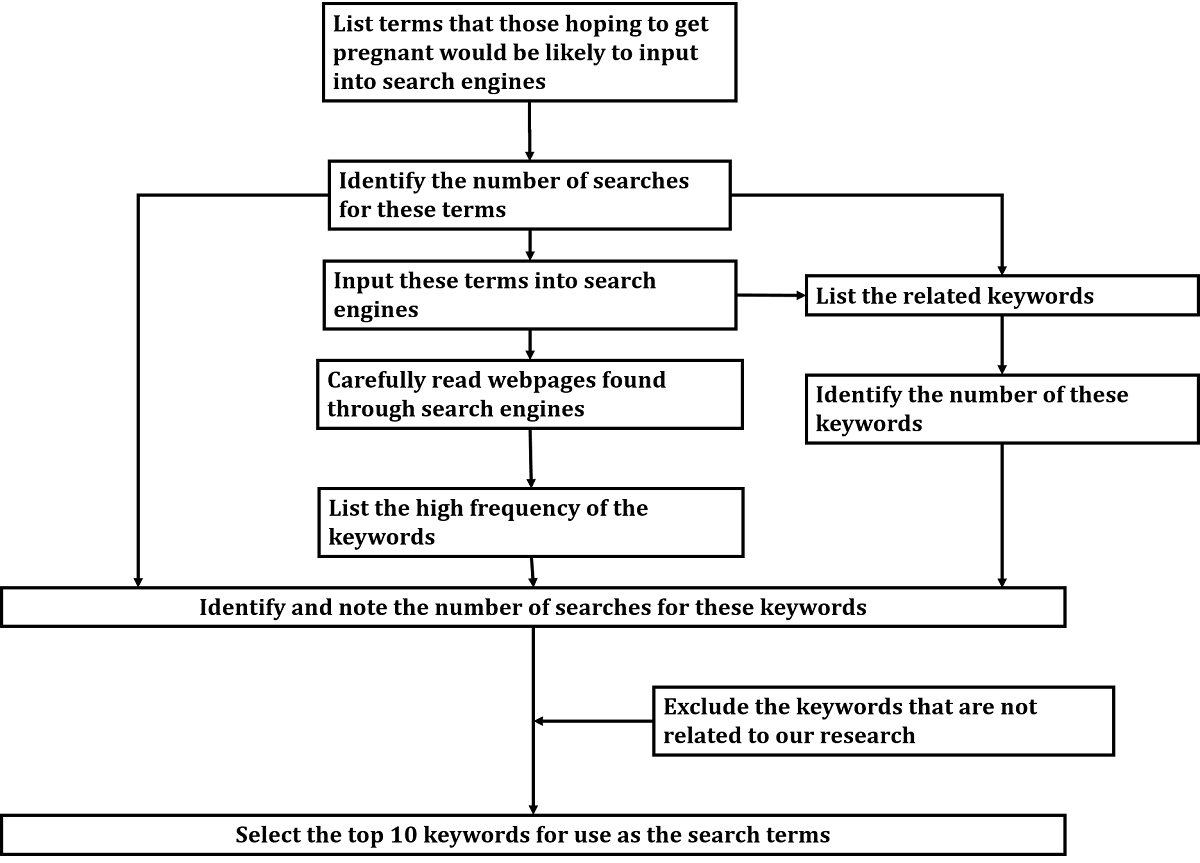
Flow diagram of the search terms generation procedure.

A flow diagram depicting the search terms generation procedure is presented in Figure 1. The search terms were determined using the following procedure. Few studies have used content analysis to examine online information about the lifestyle factors associated with reduced fertility. Although English speakers frequently refer to “fertility,” Japanese speakers do not commonly use the word “ninyousei” (fertility). Thus, deriving search terms from the previous literature was difficult. The main search terms thought to be used by people who are trying to conceive were derived in the following manner. First, we listed terms such as “ninshin” (pregnancy), “ninkatsu” (trying to conceive), and “funin” (infertility) to capture the most common and basic keywords that those hoping to get pregnant would be likely to input into search engines. We identified the number of searches for each of these keywords using a keyword search calculation tool [44]. Although both Google (Keyword Planner) and Yahoo (keyword advice tool) have tools to check the number of monthly searches, we were not able to use these services because we would need to register as a corporation to do so. Therefore, we used the keyword search calculation tool produced by Devo Inc [44]. This keyword search tool provides the total number of searches in one month for the search term, as well as the total number of searches for the top 50 webpages, per Google Japan and Yahoo! Japan [44]. After carefully reading websites found through Google Japan and Yahoo! Japan, we listed additional keywords such as “bebimachi” (waiting for a baby) that people who are trying to conceive would be likely to input. Then, we described the number of searches for each keyword using the abovementioned keyword search calculation tool. The top 6 keywords in terms of the number of searches were “funin” (infertility), “ninkatsu” (trying to conceive), “ninshindekinai” (I cannot get pregnant), “akachanhosii” (hoping to have a baby), “shizenninshin” (natural conception), and “bebimachi” (waiting for a baby).

As a next step, we entered these 6 keywords into Google Japan and Yahoo! Japan and listed the related keywords shown by the search engines (eg, “ninkatsu sapurimento” [trying to conceive AND supplement]). Related keywords are words that are frequently searched in combination with the main keywords [45]. Additionally, we described the number of searches for related keywords such as “funin genin” (infertility cause) by entering the top 6 keywords into the keyword search calculation tool [44]. The objective of this study was not to examine the information available to people who have already been diagnosed with infertility or those who are receiving infertility treatment. Rather, the objective was to examine the information accessible to those who are trying to conceive or hoping to have a child in the future. Therefore, we excluded the following from the listed search phases keywords concerning specific commercial products or services, financial support for infertility, and specific risk factors for infertility such as endometriosis or azoospermia. Of the remaining keywords, the top 10 were listed using the keyword search calculation tool. Using Google Trends, we then confirmed that the number of searches for the top 10 search terms did not represent a surge, compared with the history of searches over the last 10 years [46].

At the end of this process, the final search was performed using the following keywords: “ninkatsu” (trying to conceive), “ninshinshitai” (hoping to get pregnant), “funin” (infertility), “bebimachi” (waiting for a baby), “funin genin” (infertility cause), “kodomohosii” (hoping to have a child), “akachanhosii” (hoping to have a baby), “ninshindekinai” (I cannot get pregnant), “shizenninshin” (natural conception), and “huninsho genin” (infertility cause).

### Material Collection

The unit of analysis in this study was the webpages suggested by the search engines. Because websites differ considerably in size, coding entire websites could introduce biases based on size [47,48]. Moreover, it has been found that visitors rarely look though all the pages of a website [47,48]. Additionally, a previous study indicated that those who used search engines stayed on each website for a mean of only 1 minute and 9 seconds (median 37 seconds) [49]. Therefore, for each webpage suggested by the search engines, this study analyzed the major part of the website that visitors were likely to read.

**Figure 2:**
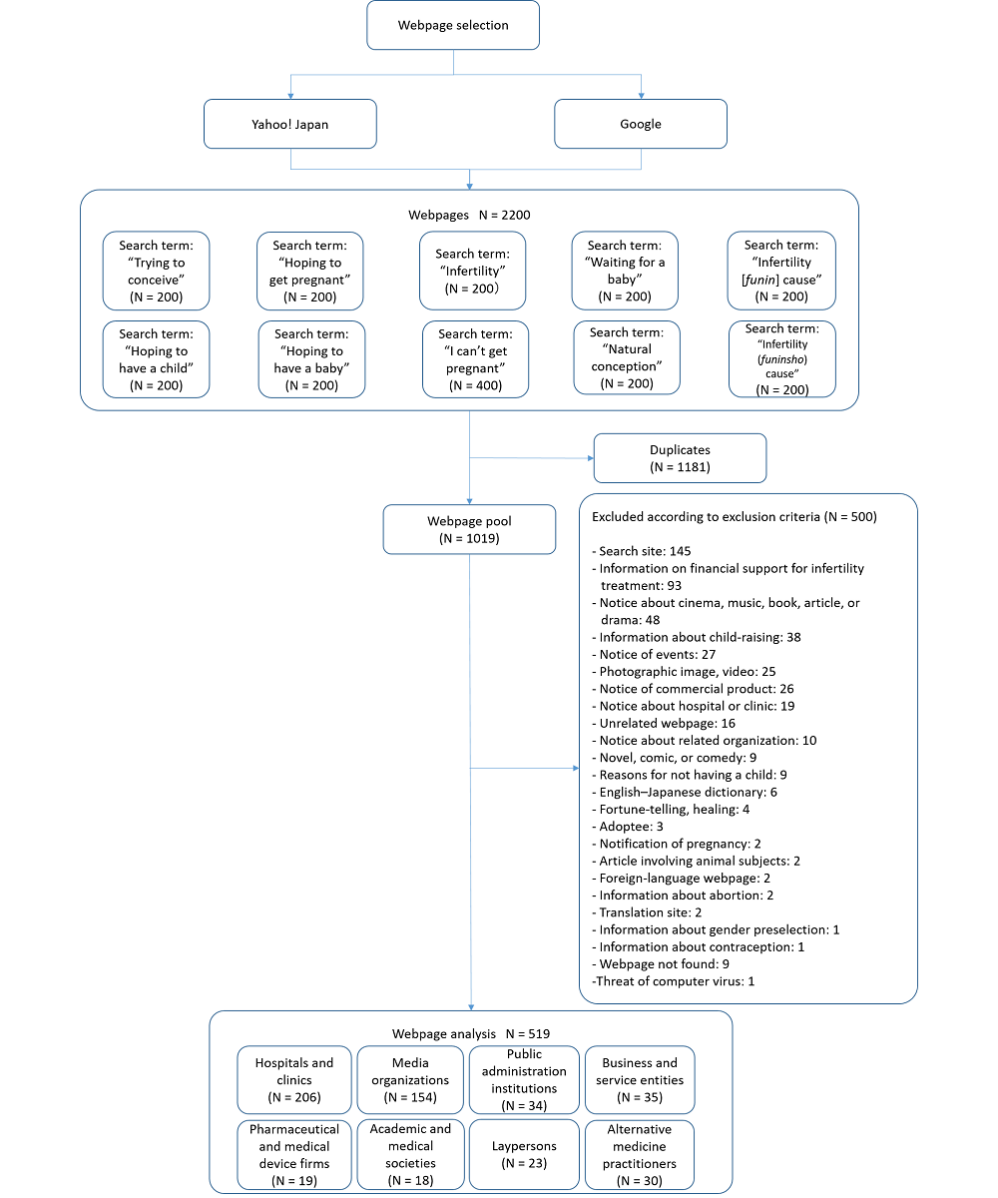
Flow diagram of the webpage selection.

The first 100 results retrieved using each search engine were collected by the first author. Each time we input search keywords or changed search engines, the search history and cookies were cleared. We conducted online searches from October 21 to November 3, 2019. The flow diagram of the webpage selection is presented in Figure 2. Of the total 2200 webpages, 1181 duplicates were excluded. Additionally, 500 webpages not directly related to the objective of this study were excluded. Ultimately, 519 webpages were included in the analysis. The URL and ranking of each result for the 2200 webpages were saved in Excel (Microsoft Corporation). The 519 webpages to be analyzed were stored as PDFs and, if that was impossible, they were copied and pasted into Word (Microsoft Corporation).

### Coding Guidelines and Procedures

There are no current clinical practice guidelines in Japan regarding lifestyle factors associated with reduced fertility. We created a list of lifestyle factors associated with reduced fertility by reviewing information from the Japan Society of Obstetrics and Gynecology [50], Ministry of Health, Labour and Welfare [51], Cochrane Library [5], British Fertility Society [52], American Society for Reproductive Medicine [53], and previous research [10-27]. This list was then reviewed by two obstetrician-gynecologists. Through discussion, we reached consensus to include the following 9 lifestyle factors associated with reduced fertility: (1) sexually transmitted diseases, (2) psychological stress, (3) cigarette smoking, (4) alcohol use, (5) nutrition and diet, (6) physical activity and exercise, (7) underweight, (8) overweight and obesity, and (9) environmental pollutants. These lifestyle factors and the major references supporting their inclusion are summarized in Table 1.

Each webpage was categorized according to the author’s professional expertise: hospitals and clinics, media organizations, public administration institutions, business and service entities, academic and medical societies, pharmaceutical and medical device firms, alternative medicine practitioners, or laypersons. Hospitals and clinics indicated that the content appeared on the website of a hospital or clinic, including blogs written by clinicians. Media organizations indicated that the content appeared on the website of a mass media organization such as a newspaper, magazine, or news site. Public administration institutions indicated that the content appeared on the website of a public organization such as the government, a municipality, public health care center, or specialized public consultation support center. Business and service entities indicated that the content appeared on the website of an enterprise such as a marriage support service company, bridal company, recruiting company, children’s goods retail business, cooking school, counseling organization, architectural firm, stationery company, or hospital search service company. Academic and medical societies indicated that the content appeared on the website of a medical society such as an association of physicians or medical institutes. Pharmaceutical and medical device firms indicated that the content appeared on the website of a pharmaceutical company, medical device firm, or pharmacy. Alternative medicine practitioners indicated that the content appeared on the website of a practitioner of alternative medicine such as osteopathy, herbal medicine Kampo, acupuncture, moxibustion, or yoga or on the website of a health food company. Laypersons meant that the content was written by persons who were currently trying or had previously tried to conceive, including patients.

**Table 1:** Lifestyle factors and major references supporting their inclusion.

Lifestyle factor and reference		Relevant sex		
		Male	Female	Unknown
**Sexually transmitted diseases**				
	Cochrane Library			x
	Japan Society of Obstetrics and Gynecology		x	
	American Society for Reproductive Medicine	x	x	
	Ministry of Health, Labour, and Welfare	x	x	
**Psychological stress**				
	Japan Society of Obstetrics and Gynecology	x		
	American Society for Reproductive Medicine		x	
	Ministry of Health, Labour, and Welfare	x		
**Cigarette smoking**				
	Cochrane Library	x	x	
	Japan Society of Obstetrics and Gynecology		x	
	British Fertility Society			x
	American Society for Reproductive Medicine	x	x	
**Alcohol use**				
	Cochrane Library	x	x	
	British Fertility Society			x
	American Society for Reproductive Medicine			x
**Nutrition and diet**				
	Cochrane Library	x	x	
**Physical activity and exercise**				
	Cochrane Library	x	x	
**Underweight**				
	Cochrane Library	x	x	
	Japan Society of Obstetrics and Gynecology		x	
	Ministry of Health, Labour, and Welfare		x	
**Overweight and obesity**				
	Cochrane Library	x	x	
	Japan Society of Obstetrics and Gynecology		x	
	British Fertility Society			x
	American Society for Reproductive Medicine		x	
	Ministry of Health, Labour, and Welfare	x	x	
**Environmental pollutants**				
	Cochrane Library	x	x	
	American Society for Reproductive Medicine			x

We created coding rules for the selected lifestyle factors. These coding guidelines are summarized in Table 2. Our coding included expressions directly related to the selected lifestyle factors associated with reduced fertility. However, expressions exclusively concerning the influence of these factors on fetuses or babies were excluded.

**Table 2:** Coding guidelines.

Lifestyle factor	Description
Sexually transmitted diseases	Content directly related to sexually transmitted diseases, including *Chlamydia trachomatis* and gonorrhea, is included. Additionally, content related to sexually transmitted disease screening or examination for causes of infertility is included.
Psychological stress	Content related to psychological stress in daily life or occupational life is included. Additionally, content related to psychological stress caused by infertility is included.
Cigarette smoking	Content directly related to cigarette smoking is included. Additionally, expressions concerning quitting smoking are included.
Alcohol use	Content directly related to alcohol is included. Additionally, expressions concerning alcohol drinking are included.
Nutrition and diet	Content directly related to nutrition and diet is included. Additionally, expressions concerning nutrient factors are included. Content only related to nutrient factors for the purpose of marketing (eg, information about supplement only) is excluded.
Physical activity and exercise	Content directly related to physical activity and exercise is included. Additionally, expressions concerning obesity prevention and exercise in regular life are included.
Underweight	Content directly related to underweight is included. Additionally, expressions concerning precipitous weight loss, dieting, and underweight as indicated by body mass index are included.
Overweight and obesity	Content directly related to overweight and obesity is included. Additionally, expressions concerning precipitous weight gain, and overweight and obesity as indicated by body mass index are included.
Environmental pollutants	Content directly related to environmental pollutants is included. Additionally, expressions concerning environmental hormones are included.

We analyzed the textual data on the websites retrieved using the search terms described above. First, we read the text carefully. Second, all data were coded on the selected lifestyle factors, author’s professional expertise, and sex of the target audience (ie, information for men, women, or both). Finally, data from all webpages were assembled and pooled in Microsoft Excel. When information on lifestyle factors was provided, the code of 1 was assigned. When no information on lifestyle factors was provided, we assigned the code of 0. The URL and title of each webpage were saved as a reference during the data analysis. Because multiple types of lifestyle factors could be listed on a single website, instead of calculating the number of webpages mentioning a particular lifestyle factor, we calculated the number of mentions (codes) for each selected lifestyle factor associated with reduced fertility.

### Interrater Reliability

Approximately 20% of the final dataset (100/519, 19.3%) was evaluated by two independent, blinded raters (RY and EF) to examine interrater reliability. Using the coding guidelines created by the first author of this study (RY), EF was instructed on applying the coding system in a training session that lasted about 1 hour. In a pilot test phase, the two raters applied the coding system to 10 webpages randomly selected from the full sample. No problems were identified during this phase. After a formal reliability assessment phase was completed, the first author (RY) calculated the interrater reliability index.

### Statistical Analysis

To assess interrater reliability, the Gwet agreement coefficient (AC1) statistic, which is less affected by prevalence compared with the Cohen kappa [54], was used to assess interrater agreement of the coding. We also conducted a test to assess differences in frequency between the two most common types of professional expertise. In addition, we compared differences between male-specific and female-specific information on lifestyle factors associated with reduced fertility by categorizing the information into two groups: (1) information for men plus information for both men and women and (2) information for women plus information for both men and women. Lifestyle factors that could not be classified in this way were treated as missing in the above tests. Differences in the author’s professional expertise, lifestyle factors, and sex of the target audience were assessed using count data analyzed by using the chi-square test and Fisher exact test. Statistical significance was set at *P*<.05 for all comparisons. Analyses were performed using R for Windows version 3.5.1 (R Foundation for Statistical Computing).

### Ethical Considerations

This study was granted an exemption from the requirement of ethics approval by the ethical review committee at the Graduate School of Medicine, The University of Tokyo, because we aimed to analyze online information, meaning that this study was not medical research involving human subjects, and because the authors had no conflicts of interest related to this study.

## Results

### Distributions of Author’s Professional Expertise

The assignment of all codes ranged from 0 to 9 (mean 1.017) per page. The assignment of codes ranged from 0 to 8 (mean 1.058) for hospitals and clinics, from 0 to 8 (mean 0.974) for media organizations, from 0 to 9 (mean 1.147) for public administration institutions, from 0 to 6 (mean 1.029) for business and service entities, from 0 to 6 (mean 1.389) for academic and medical societies, from 0 to 7 (mean 2.105) for pharmaceutical and medical device firms, from 0 to 3 (mean 0.367) for alternative medicine practitioners, and from 0 to 3 (mean 0.391) for laypersons. Of the webpages retrieved, 0 codes were assigned to 60.7% (315/519). The number of webpages for which 0 codes were assigned was 53.4% (110/206) for hospitals and clinics, 66.2% (102/154) for media organizations, 68% (23/34) for public administration institutions, 60% (21/35) for business and service entities, 50% (9/18) for academic and medical societies, 53% (10/19) for pharmaceutical and medical device firms, 77% (23/30) for alternative medicine practitioners, and 74% (17/23) for laypersons.

The distributions of the webpage author’s professional expertise are summarized in Multimedia Appendix 1. Of the webpages retrieved, 39.7% (206/519) were produced by hospitals and clinics, 29.7% (154/519) by media organizations, 6.6% (34/519) by public administration institutions, 6.7% (35/519) by business and service entities, 5.8% (30/519) by alternative medicine practitioners, 4.4% (23/519) by laypersons, 3.7% (19/519) by pharmaceutical or medical device firms, and 3.5% (18/519) by academic and medical societies. Of the codes assigned for the examined lifestyle factors associated with reduced fertility, 41.2% (218/528) were published by hospitals and clinics, 28.4% (150/528) by media organizations, 7.6% (40/528) by pharmaceutical and medical device firms, 7.4% (39/528) by public administration institutions, 6.8% (36/528) by business and service entities, 4.7% (25/528) by academic and medical societies, 2.1% (11/528) by alternative medicine practitioners, and 1.7% (9/528) by laypersons.

### Interrater Reliability

A review of the coding by the two independent, blinded raters on the randomly selected subsample of 100 webpages revealed that the interrater agreement was excellent. The two raters agreed on the assignment of codes in 98.00% (3528/3600) of coding instances. The Gwet AC1 statistic for the assignment of codes ranged from 0.969 to 1.000 (mean AC1 0.985) for the author’s professional expertise, from 0.957 to 0.992 (mean AC1 0.979) for lifestyle factors, and from 0.950 to 0.986 (mean AC1 0.978) for the sex of the target audience. For the data analysis in this study, we used the coding of the first author.

### Distribution of Lifestyle Factors

Of the coding instances concerning lifestyle factors associated with reduced fertility, 20.1% (106/528) related to psychological stress and 18.8% (99/528) related to sexually transmitted diseases. Together, codes on these two lifestyle factors accounted for 38.9% (205/528) of all lifestyle factor-coding instances. Websites referring to the associations between reduced fertility and nutrition and diet, cigarette smoking, underweight, physical activity and exercise, overweight and obesity, alcohol use, and environmental pollutants were relatively rare.

### Distribution of Lifestyle Factors by Author’s Professional Expertise

**Figure 3:**
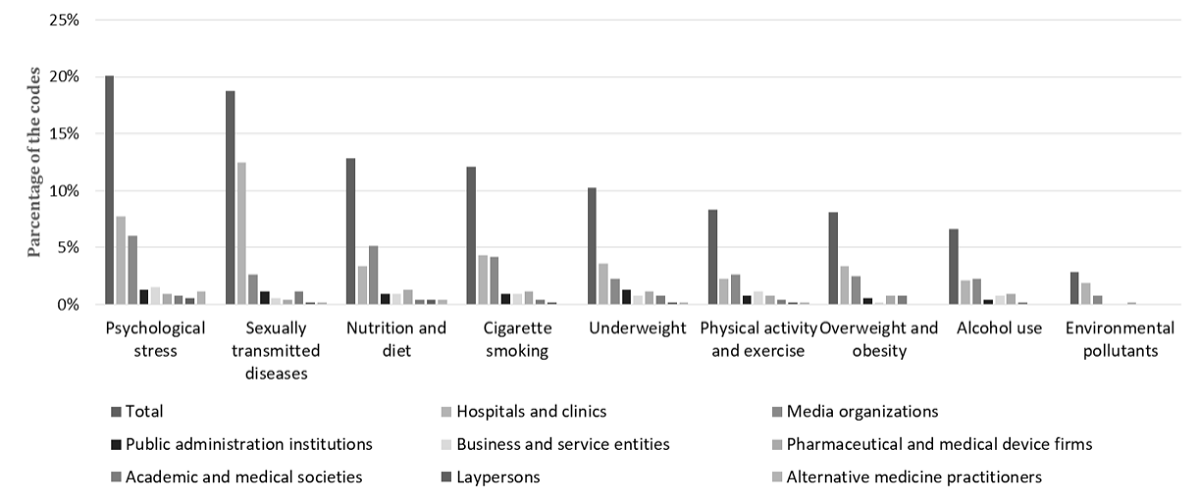
Distribution of codes for lifestyle factors associated with reduced fertility by the author’s professional expertise.

Figure 3 illustrates the distribution of codes for the examined lifestyle factors associated with reduced fertility by author’s professional expertise. Of the coding instances referring to the association between reduced fertility and sexually transmitted diseases, 67% (66/99) were published by hospital or clinics and 14% (14/99) were published by media organizations. We assessed differences in the frequency of each lifestyle factor code between the two most common types of author’s professional expertise (ie, hospitals/clinics and media organizations) using the chi-square test and Fisher exact test (Table 3). The results of these tests showed that the association between sexually transmitted diseases and reduced fertility was mentioned more frequently by hospitals and clinics than by media organizations (*P*<.001). In contrast, of the coding instances referring to the association between nutrition and diet and reduced fertility, 26% (18/68) were published by hospitals and clinics and 40% (27/68) were published by media organizations; the association between nutrition and diet and reduced fertility was significantly more frequently mentioned by the media than by hospitals and clinics (*P*=.008). The other lifestyle factors associated with reduced fertility did not show statistically significant differences between hospitals/clinics and media organizations.

**Table 3:** Lifestyle factors and their associations with webpage author’s professional expertise for hospitals or clinics and media organizations.

Lifestyle factor		Author’s professional expertise, n (%)		Chi-square	*df* ^a^	*P* value^b^
		Hospitals and clinics (n=218)	Media organizations (n=150)			
**Sexually transmitted diseases**				21.7	1	<.001^c^
	Number of webpages with codes	66 (30.3)	14 (9.3)			
	Number of webpages without codes	152 (69.7)	136 (90.7)			
**Psychological stress**				0.2	1	.64^c^
	Number of webpages with codes	41 (18.8)	32 (21.3)			
	Number of webpages without codes	177 (81.2)	118 (78.7)			
**Cigarette smoking**				1	1	.31^c^
	Number of webpages with codes	23 (10.6)	22 (14.7)			
	Number of webpages without codes	195 (89.4)	128 (85.3)			
**Alcohol use**				0.9	1	.35^c^
	Number of webpages with codes	11 (5.0)	12 (8.0)			
	Number of webpages without codes	207 (95.0)	138 (92.0)			
**Nutrition and diet**				7	1	.008^c^
	Number of webpages with codes	18 (8.3)	27 (18.0)			
	Number of webpages without codes	200 (91.7)	123 (82.0)			
**Physical activity and exercise**				1.4	1	.23^c^
	Number of webpages with codes	12 (5.5)	14 (9.3)			
	Number of webpages without codes	206 (94.5)	136 (90.7)			
**Underweight**				0	1	.96^c^
	Number of webpages with codes	19 (8.7)	12 (8.0)			
	Number of webpages without codes	199 (91.3)	138 (92.0)			
**Overweight and obesity**				0	1	>.99^c^
	Number of webpages with codes	18 (8.3)	13 (8.7)			
	Number of webpages without codes	200 (91.7)	137 (91.3)			
**Environmental pollutants**				—	—	.42^d^
	Number of webpages with codes	10 (4.6)	4 (2.7)			
	Number of webpages without codes	208 (95.4)	146 (97.3)			

^a^Degrees of freedom.

^b^*P* values compare media organizations with hospitals and clinics.

^c^Chi-square test.

^d^Fisher exact test.

### Distribution of Lifestyle Factors by the Sex of the Target Audience

**Figure 4:**
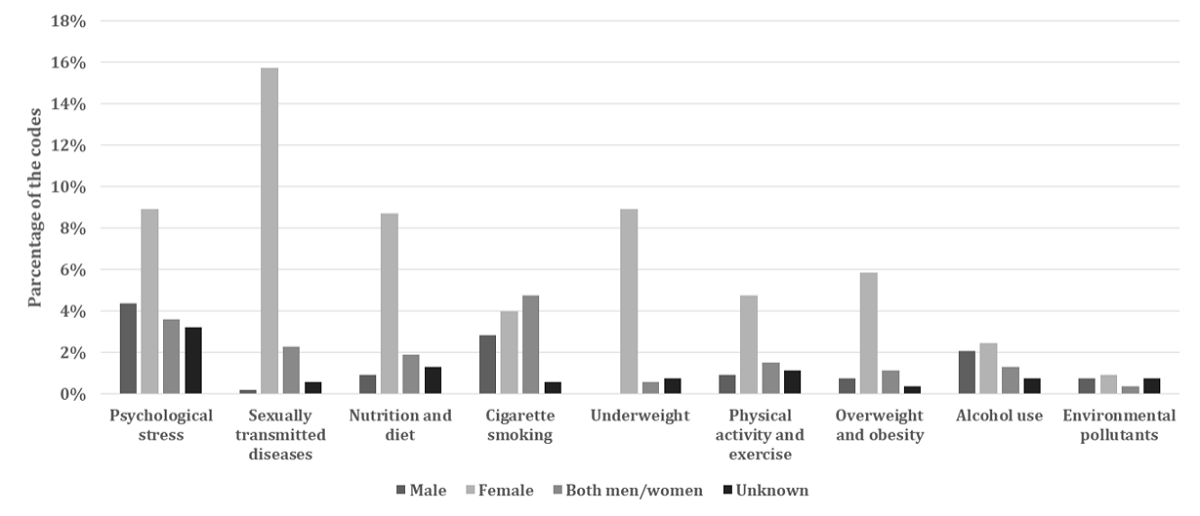
Distribution of codes for lifestyle factors associated with reduced fertility by the sex of the target audience.

Figure 4 illustrates the distribution of codes for lifestyle factors associated with reduced fertility by the sex of the target audience. Of the total lifestyle factor-coding instances, 12.9% (68/528) were about men’s lifestyle factors, 60.2% (318/528) were about women’s lifestyle factors, and 17.4% (92/528) were about both women’s and men’s lifestyle factors (see Multimedia Appendix 2). Across all examined types of lifestyle factors, female-specific information was observed more frequently than was male-specific information. Information referring to psychological stress, sexually transmitted diseases, nutrition and diet, underweight, physical activity and exercise, and overweight and obesity was significantly more frequently directed toward women than toward men (Table 4; psychological stress: *P*=.002, sexually transmitted diseases: *P*<.001, nutrition and diet: *P*<.001, underweight: *P*<.001, physical activity and exercise: *P*<.001, overweight and obesity: *P*<.001).

**Table 4:** Lifestyle factors and their associations with the sex of the target audience.

Lifestyle factor		Sex of target audience		Chi-square	*df* ^b^	*P* value^c^
		Male^a^, n (%)	Female^a^, n (%)			
**Sexually transmitted diseases (n=108)**				121.5	1	<.001^d^
	Number of webpages with codes	13 (12.0)	95 (88.0)			
	Number of webpages without codes	95 (88.0)	13 (12.0)			
**Psychological stress (n=108)**				9.8	1	.002^d^
	Number of webpages with codes	42 (38.9)	66 (61.1)			
	Number of webpages without codes	66 (61.1)	42 (38.9)			
**Cigarette smoking (n=86)**				0.6	1	.45^d^
	Number of webpages with codes	40 (46.5)	46 (53.5)			
	Number of webpages without codes	46 (53.5)	40 (46.5)			
**Alcohol use (n=38)**				0.1	1	.82^d^
	Number of webpages with codes	18 (47.4)	20 (52.6)			
	Number of webpages without codes	20 (52.6)	18 (47.4)			
**Nutrition and diet (n=71)**				45.1	1	<.001^d^
	Number of webpages with codes	15 (21.1)	56 (78.9)			
	Number of webpages without codes	56 (78.9)	15 (21.1)			
**Physical activity and exercise (n=46)**				15.7	1	<.001^d^
	Number of webpages with codes	13 (28.3)	33 (71.7)			
	Number of webpages without codes	33 (71.7)	13 (28.3)			
**Underweight (n=53)**				—	—	<.001^e^
	Number of webpages with codes	3 (5.7)	50 (94.3)			
	Number of webpages without codes	50 (94.3)	3 (5.7)			
**Overweight and obesity (n=47)**				28.8	1	<.001^d^
	Number of webpages with codes	10 (21.3)	37 (78.7)			
	Number of webpages without codes	37 (78.7)	10 (21.3)			
**Environmental pollutants (n=13)**				—	—	>.99^e^
	Number of webpages with codes	6 (46.2)	7 (53.8)			
	Number of webpages without codes	7 (53.8)	6 (46.2)			

^a^We categorized the information into two groups: (1) information for men plus information for both men and women and (2) information for women plus information for both men and women. Lifestyle factors that could not be classified in this way were treated as missing.

^b^Degrees of freedom.

^c^*P* values compare the information for men with the information for women.

^d^Chi-square test.

^e^Fisher exact test.

## Discussion

### Principal Findings

This study quantitatively examined online information on the lifestyle factors associated with reduced fertility. The main findings of the study confirm that a large proportion of the information on lifestyle factors associated with reduced fertility was disseminated by hospitals, clinics, and the media. Furthermore, the findings show that the frequencies of descriptions provided by different entities varied by the particular lifestyle factor examined.

### Distribution of Author’s Professional Expertise

In terms of the professional expertise of the author publishing online information on lifestyle factors associated with reduced fertility, following the categorization of professional expertise described in the Methods section, our study demonstrated that, of the codes assigned for the examined lifestyle factors associated with reduced fertility, 41.2% (218/528) were published by hospitals and clinics, 28.4% (150/528) by media organizations, and 1.7% (9/528) by laypersons. This may be because of the strategy of search engine optimization used by hospitals and clinics to make their websites appear higher in user lists of search results generated by the search engines. This may also be because lifestyle factors associated with reduced fertility are discussed in the context of infertility examinations or treatments. The large number of references to lifestyle factors associated with reduced fertility by media organizations may be associated with the mass media coverage of the public debate [55] after the Japanese government’s proposal for the need for fertility education in 2013 [28]. Conversely, the low number of references to lifestyle factors associated with reduced fertility by laypersons may be associated with perceived stigma around infertility [56]: Laypersons may avoid discussing these factors because of concerns about stigma related to infertility or not being able to get pregnant. In contexts where the desire for children is generally regarded as the social norm, higher levels of stigma consciousness may be associated with reductions in disclosure of one’s own infertility [56]. Those who disclose their infertility may also have more negative social experiences associated with their infertility in such contexts [56] and consequently avoid further disclosing their infertility in the future. Therefore, the stigma around infertility may explain why laypersons were found to rarely mention lifestyle factors associated with reduced fertility in this study.

### Distribution of Lifestyle Factors

Psychological stress and sexually transmitted diseases were the two most frequently mentioned lifestyle factors associated with reduced fertility. In contrast, the associations between reduced fertility and cigarette smoking, alcohol use, physical activity and exercise, underweight, and overweight and obesity were less frequently discussed. Our findings are consistent with those of a previous study showing that people lack a general understanding of fertility, including the associations between reduced fertility and cigarette smoking and overweight and obesity [28]. In 2017, the National Health and Nutrition Survey demonstrated that (1) 40% to 50% of the reproductive-aged population in Japan drank alcohol [57], (2) more than one-quarter of reproductive-aged men were obese [57], (3) 10% to 20% of reproductive-aged women were underweight [57], and (4) approximately 20% of the population smoked cigarettes [57]. Despite this situation, the associations between reduced fertility and alcohol consumption of both men and women, male overweight and obesity, female underweight, and cigarette smoking of both men and women were mentioned relatively infrequently on the websites analyzed in this study. These lifestyle factors should be discussed more frequently. We also believe that public initiatives for the reproductive-aged population and a fertility-related information strategy should be established in Japan. Several exemplary websites with evidence-based information exist that could serve as a model for these changes [58,59].

### Distribution of Lifestyle Factors by Author’s Professional Expertise

Our research has revealed that associations between reduced fertility and both psychological stress and sexually transmitted diseases were more frequently discussed by hospitals and clinics than by the other types of webpage author. This may be because lifestyle factors associated with reduced fertility are discussed in the context of infertility distress regarding infertility examinations or treatments [32,60]. We also found that the association between reduced fertility and nutrition and diet was more frequently discussed by the media than by hospitals and clinics. This may be because lifestyle factors associated with reduced fertility are presented less as health-oriented information, with media outlets orienting health messages toward entertainment.

Providing treatment is the main role of hospitals and clinics, and these entities may use their websites as a tool for patient acquisition. However, for individuals seeking fertility information on the internet because they are trying to conceive, the content of hospital and clinic websites may be a major source of information on fertility. This idea is supported by the finding that hospital and clinic websites rank higher in lists of search results when users use web search engines. Hence, various kinds of information provided by hospitals and clinics may be beneficial for people who are trying to conceive. In previous work, scholars have recommended that, despite the pressures of a competitive environment, hospitals and clinics offering fertility treatment should present educational information in an ethically balanced manner [61]. The same scholars have also recommended that medical experts lead the way for best practices among doctors by creating guidelines regarding the provision of online information [61]. We additionally recommend the ethically balanced presentation of information on fertility in the media. Media websites should incorporate benchmarks for collaboration between experts and the media.

### Distribution of Lifestyle Factors by the Sex of the Target Audience

We found that there were fewer mentions of lifestyle factors associated with reduced fertility for men than for women. This may be because (1) men are less likely to seek information on infertility [32], (2) male and female infertility is highly stigmatized, and (3) men have a relatively low level of knowledge regarding male infertility [62]. Regarding stigma around male infertility, reproductive health is often regarded as a women’s issue [31]. This may be because fertility treatment has mainly focused on women’s bodies, although male factors also contribute to infertility [31]. Likewise, previous work indicates that infertility tends to be regarded as a women’s issue in Japan [63]. Message senders may be influenced by this social norm, and they may therefore convey messages regarding infertility in a way that is consistent with this topic being a women’s issue. Conversely, male infertility may be related to stigma stemming from ideas about masculinity that many men consider to be a social norm [60,64]. Attempts to hide stigmatized conditions often lead to delays in information-seeking behavior [65]. Therefore, it is important that published discussions on the lifestyle factors associated with reduced fertility include information for both men and women.

### Infertility and Stigma

As mentioned above, infertility may harm the self-esteem of people who are trying to conceive because of its latently stigmatizing nature [56]. People with a stigmatized condition tend to use the internet to seek health information more often compared with people with nonstigmatized conditions [65]. Those who experience infertility may feel a sense of isolation and be less likely to seek social support because of reduced self-esteem [56]. Advantages of the internet include user anonymity, optional disclosure, and the lack of geographical barriers [66]. These advantages could mitigate the threat of social stigma and distress around disclosure, providing much needed information on fertility. It is also important to address online discussion boards in terms of informational, emotional, and appraisal support [66]. Moreover, because the internet may be a good public education tool for people with stigmatized conditions [65], we recommend the use of online media for fertility-related education [67].

### Limitations

This study has several limitations. First, we limited our examination to online information, which does not capture all circulating public messages. Television broadcasts and print newspapers and magazines may also be widely used sources of information about lifestyle factors associated with reduced fertility. Second, although a substantial number of websites (n=519) were retrieved for this analysis, availability, accessibility, and time limitations made it unfeasible to analyze all relevant sites. Third, despite the fact that we selected search terms based on related words indicated by the selected search engines, these search terms may have reflected our own biases. For example, it is possible that search terms were relatively easy for women to use. However, because men were less likely to seek information regarding infertility [32], the webpages for women should include information for men. Fourth, it is possible that the selected lifestyle factors associated with reduced fertility may also have reflected our biases; however, this study examined several publications from academic societies, Cochrane Library, and previous studies to determine which lifestyle factors to investigate. For example, age was not treated as a lifestyle factor associated with reduced fertility in this study because Cochrane Library did not directly consider age when defining lifestyle factors that may both influence fertility and affect the chances of a healthy, live birth [5]. Additionally, a recent study found that most of the Japanese reproductive-aged population (60% to 70%) were knowledgeable about the association between age and reduced fertility [28]. Therefore, this study did not include age in the analysis. However, age should be explored as a potential factor associated with reduced fertility in future research. Fifth, when identifying lifestyle factors associated with reduced fertility, we checked for critical threshold levels for the contribution of particular factors, but study designs, outcomes, and sample sizes varied across the examined studies. Because there are presently no guidelines concerning lifestyle factors associated with reduced fertility in any country, we could not identify unified critical thresholds for judging the impact of each lifestyle factor on reduced fertility. Sixth, we did not evaluate the accuracy of the information presented on the webpages. It is possible that some websites provided accurate information and others provided incorrect information. The accuracy of information provided on this topic should be explored in future research. Seventh, our study was conducted via online search at the end of October 2019. Although we confirmed that the number of searches for the top 10 most frequent main search terms did not represent a surge, we did not explore how the number and frequency of search results concerning the examined lifestyle factors associated with reduced fertility changed over the time. Finally, our analysis was restricted to Japanese-language online information, which may limit its generalizability to other contexts. However, considering context is crucial when examining media messages, which is an advantage of focusing the analysis on Japanese-language websites [35]. Although there are limitations to the study, to our knowledge, this study is the first to examine online information on the lifestyle factors associated with reduced fertility using quantitative content analysis.

### Conclusions

In terms of the webpage author’s professional expertise, authors from hospitals, clinics, and the media relatively frequently mentioned lifestyle factors associated with reduced fertility, whereas laypersons mentioned them relatively rarely. Regarding the specific lifestyle factors associated with reduced fertility, psychological stress and sexually transmitted diseases were more frequently discussed compared with the other factors. The association between reduced fertility and sexually transmitted diseases was more frequently discussed by hospitals and clinics than by the media. Conversely, the association between reduced fertility and nutrition and diet was significantly more frequently mentioned by the media than by hospitals and clinics. With regard to the sex of the target audience, male lifestyle factors were less frequently discussed than were female lifestyle factors. The authors of fertility-related websites should more frequently mention information on lifestyle factors associated with reduced fertility overall, moving beyond only psychological stress and sexually transmitted diseases to also discuss nutrition and diet, cigarette smoking, underweight, physical activity and exercise, overweight and obesity, alcohol use, and environmental pollutants. These authors should also make an effort to provide specific information on men’s lifestyle factors.

## Appendix

Multimedia Appendix 1 Distribution of lifestyle factor codes by webpage author’s professional expertise.

Multimedia Appendix 2 Distribution of lifestyle factor codes by the author’s professional expertise and the sex of the target audience.

## Abbreviations

AC1: Gwet agreement coefficient 1
