# Supplementary material for: Online Japanese-Language Information on Lifestyle Factors Associated With Reduced Fertility: Content Analysis
Source: J Med Internet Res. 2020 Aug 25;22(8):e19777. doi: 10.2196/19777 (PMC7479583; doi:10.2196/19777)
Supplement: Multimedia Appendix 1 [file jmir_v22i8e19777_app1.docx]

Multimedia Appendix 1. Distribution of lifestyle factor codes by webpage author’s professional expertise.

|  | **Author's professional expertise** | | | | | | | | | | | | | | | | | |
| --- | --- | --- | --- | --- | --- | --- | --- | --- | --- | --- | --- | --- | --- | --- | --- | --- | --- | --- |
|  | **Hospital or  clinic** | | **Media  organization** | | **Public  administration**  **institution** | | **Business**  **or  service**  **entity** | | **Academic or  medical  society** | | **Pharmaceutical or  medical  device firm** | | **Alternative  medicine**  **practitioner** | | **Layperson** | | **Total** | |
| **Total number of  webpages** | 206 | 39.7% | 154 | 29.7% | 34 | 6.6% | 35 | 6.7% | 18 | 3.5% | 19 | 3.7% | 30 | 5.8% | 23 | 4.4% | 519 |  |
|  |  |  |  |  |  |  |  |  |  |  |  |  |  |  |  |  |  |  |
| **Lifestyle factor** | **Author's professional expertise** | | | | | | | | | | | | | | | | | |
|  | **Hospital or  clinic** | | **Media  organization** | | **Public  administration**  **institution** | | **Business**  **or service**  **entity** | | **Academic**  **or  medical  society** | | **Pharmaceutical or  medical  device firm** | | **Alternative  medicine**  **practitioner** | | **Layperson** | | **Total** | |
| **Sexually transmitted diseases** | | | | | | | | | | | | | | | | | | |
|  | 66 | 30.3% | 14 | 9.3% | 6 | 15.4% | 3 | 8.3% | 6 | 24.0% | 2 | 5.0% | 1 | 9.1% | 1 | 11.1% | 99 | 18.8% |
| **Psychological stress** | | | | | | | | | | | | | | | | | | |
|  | 41 | 18.8% | 32 | 21.3% | 7 | 17.9% | 8 | 22.2% | 4 | 16.0% | 5 | 12.5% | 6 | 54.5% | 3 | 33.3% | 106 | 20.1% |
| **Cigarette smoking** | | | | | | | | | | | | | | | | | | |
|  | 23 | 10.6% | 22 | 14.7% | 5 | 12.8% | 5 | 13.9% | 2 | 8.0% | 6 | 15.0% | 0 | 0.0% | 1 | 11.1% | 64 | 12.1% |
| **Alcohol use** | | | | | | | | | | | | | | | | | | |
|  | 11 | 5.0% | 12 | 8.0% | 2 | 5.1% | 4 | 11.1% | 1 | 4.0% | 5 | 12.5% | 0 | 0.0% | 0 | 0.0% | 35 | 6.6% |
| **Nutrition and diet** | | | | | | | | | | | | | | | | | | |
|  | 18 | 8.3% | 27 | 18.0% | 5 | 12.8% | 5 | 13.9% | 2 | 8.0% | 7 | 17.5% | 2 | 18.2% | 2 | 22.2% | 68 | 12.9% |
| **Physical activity and exercise** | | | | | | | | | | | | | | | | | | |
|  | 12 | 5.5% | 14 | 9.3% | 4 | 10.3% | 6 | 16.7% | 2 | 8.0% | 4 | 10.0% | 1 | 9.1% | 1 | 11.1% | 44 | 8.3% |
| **Underweight** | | | | | | | | | | | | | | | | | | |
|  | 19 | 8.7% | 12 | 8.0% | 7 | 17.9% | 4 | 11.1% | 4 | 16.0% | 6 | 15.0% | 1 | 9.1% | 1 | 11.1% | 54 | 10.2% |
| **Overweight and obesity** | | | | | | | | | | | | | | | | | | |
|  | 18 | 8.3% | 13 | 8.7% | 3 | 7.7% | 1 | 2.8% | 4 | 16.0% | 4 | 10.0% | 0 | 0.0% | 0 | 0.0% | 43 | 8.1% |
| **Environmental pollutants** | | | | | | | | | | | | | | | | | | |
|  | 10 | 4.6% | 4 | 2.7% | 0 | 0.0% | 0 | 0.0% | 0 | 0.0% | 1 | 2.5% | 0 | 0.0% | 0 | 0.0% | 15 | 2.8% |
| **All codes** | | | | | | | | | | | | | | | | | | |
|  | 218 |  | 150 |  | 39 |  | 36 |  | 25 |  | 40 |  | 11 |  | 9 |  | 528 |  |
